# Supplementary material for: Targeting Cancer Metabolism Breaks Radioresistance by Impairing the Stress Response
Source: Cancers (Basel). 2021 Jul 27;13(15):3762. doi: 10.3390/cancers13153762 (PMC8345170; doi:10.3390/cancers13153762)
Supplement: Supplementary file 1 [file cancers-13-03762-s001.zip › cancers-1291083-supplementary.pdf]

cancers

Supplementary Materials

Targeting Cancer Metabolism Breaks Radioresistance by Impairing the Stress Response

Melissa Schwab, Katharina Thunborg, Omid Azimzadeh, Christine von Toerne, Caroline Werner, Maxim Shevtsov, Tommaso Di Genio, Masa Zdralevic, Jacques Pouyssegur, Kathrin Renner, Marina Kreutz and Gabriele Multhoff

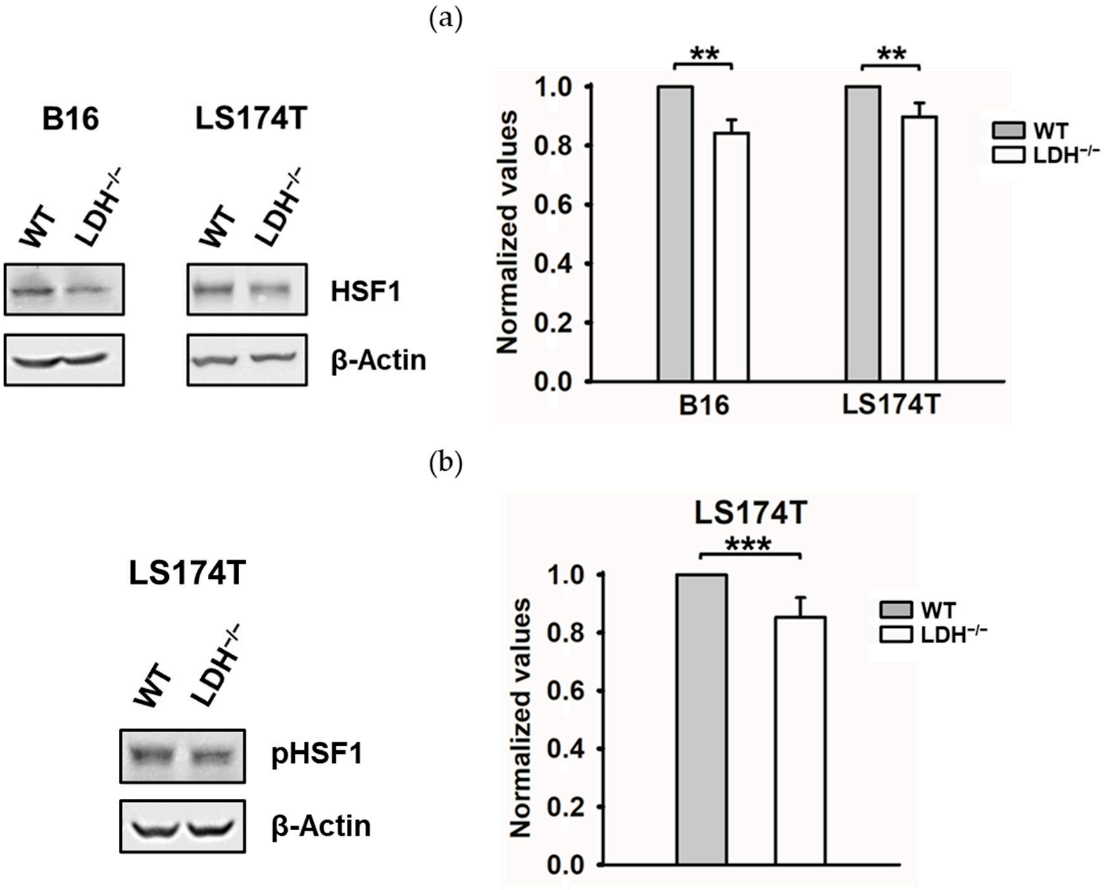

Figure S1. Heat shock factor 1 (HSF1) expression after *LDHA*/*B* double knockout (LDH$^{-/-}$). Representative immunoblot showing the cytosolic non-phosphorylated HSF1 (**a**) and phosphorylated pHSF1; (**b**) expression of B16F10 and LS174T wildtype (WT) and LDH$^{-/-}$ cells. β-Actin was used as a loading control. The quantifications of the protein expression levels are shown in the adjacent bar chart. Error bars show the standard deviations (SD) of at least three biological replicates (**: $p \leq 0.01$ and ***: $p \leq 0.001$). Full Western blot images are available in Figures S9 and S10.

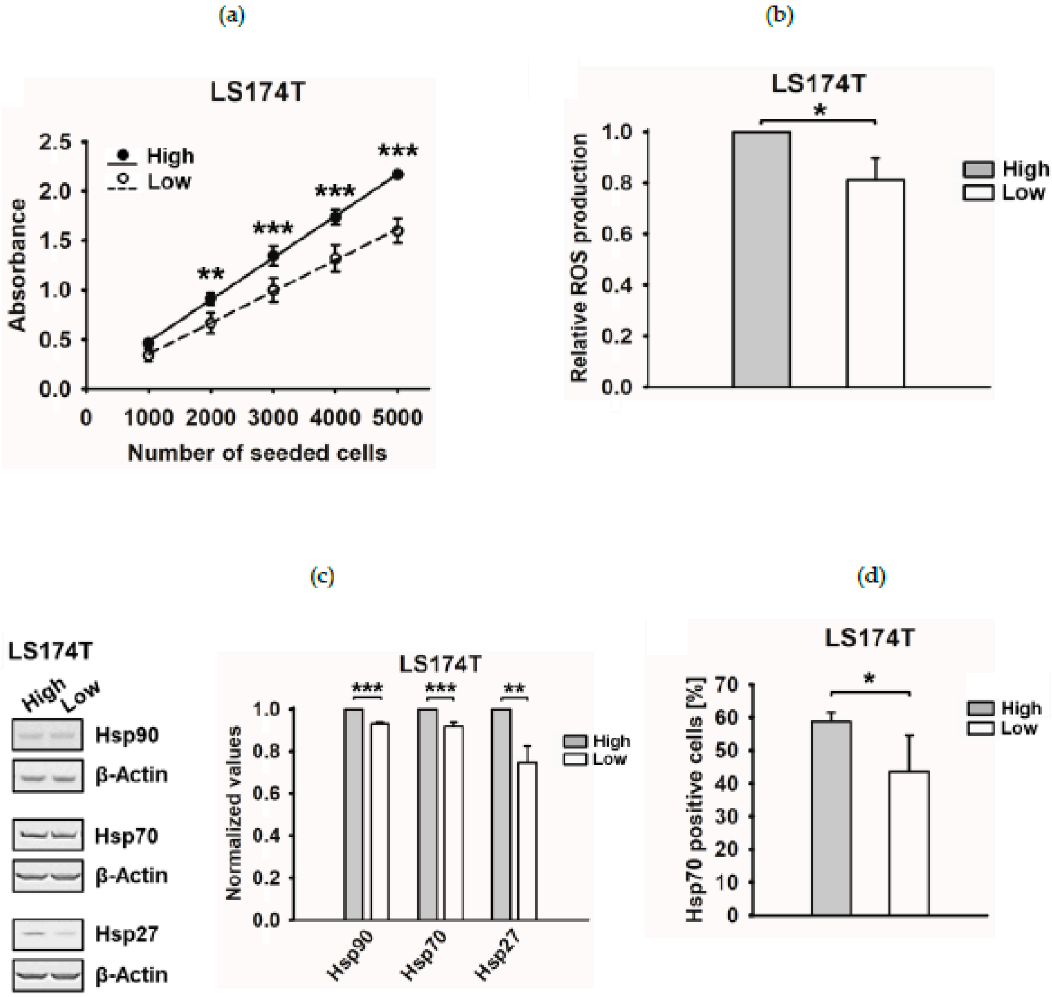

Figure S2. Glucose amount influences the proliferation capacity, reactive oxygen species (ROS) production and, thereby, the expression of the heat shock proteins (HSPs). (**a**) CCK-8 assay showing a slower proliferation in LS174T cells cultured in a medium with low glucose (Low, 1000-mg/mL glucose) compared to a high-glucose medium (High, 4500-mg/mL glucose) (**: $p \leq 0.01$ and ***: $p \leq 0.001$). (**b**) Intracellular ROS levels in LS174T cells were measured by the DCFDA assay. Error bars show the standard deviations (SD) of three biological replicates (*: $p \leq 0.05$). (**c**) Representative immunoblot showing the expression of intracellular Hsp90, Hsp70 and Hsp27 in LS174T cells cultured in a medium with a low or high glucose amount. β-Actin was used as a loading control. The quantifications of the expression levels of the HSPs are shown in the adjacent bar chart. Error bars show the standard deviation (SD) of at least three biological replicates (**: $p \leq 0.01$ and ***: $p \leq 0.001$). (**d**) Membrane-bound Hsp70 on LS174T cells cultured in a medium with a low or high glucose amount was measured by flow cytometry. The proportion of positively stained cells is shown. Error bars show the SD of four biological replicates (*: $p \leq 0.05$). Full Western blot images are available in Figure S11.

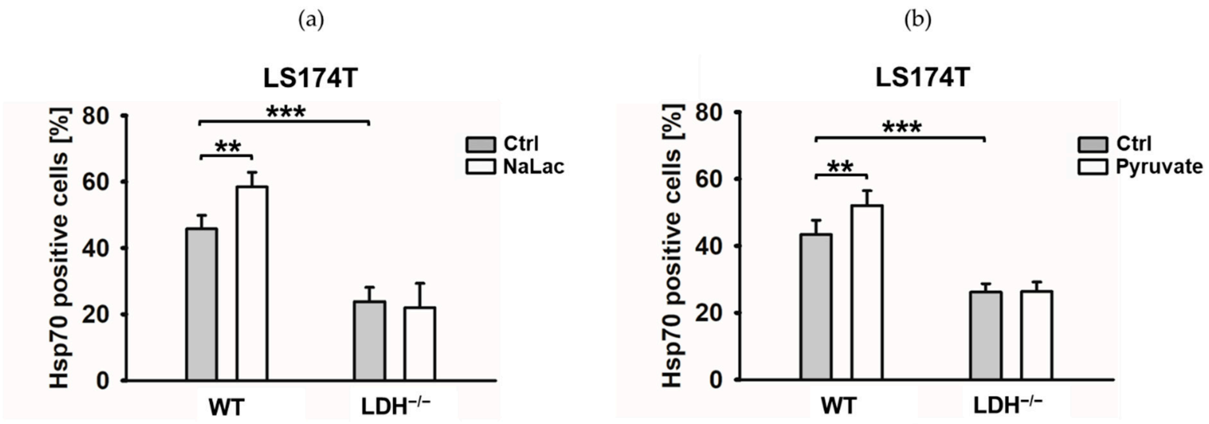

Figure S3. Lactate and pyruvate addition increases membrane-bound Hsp70 in the wiltype (WT) but not in *LDHA*/*B* double knockout (LDH$^{-/-}$) tumor cells. LS174T WT and LDH$^{-/-}$ were treated with 15-mM sodium lactate (NaLac) (**a**) and 15-mM sodium pyruvate (**b**) for 6 h. Membrane-bound Hsp70 was analyzed by flow cytometry. The proportion of positively stained cells is shown. Error bars show the standard deviations (SD) of four biological replicates (**: $p \leq 0.01$ and ***: $p \leq 0.001$). Ctrl: untreated cells.

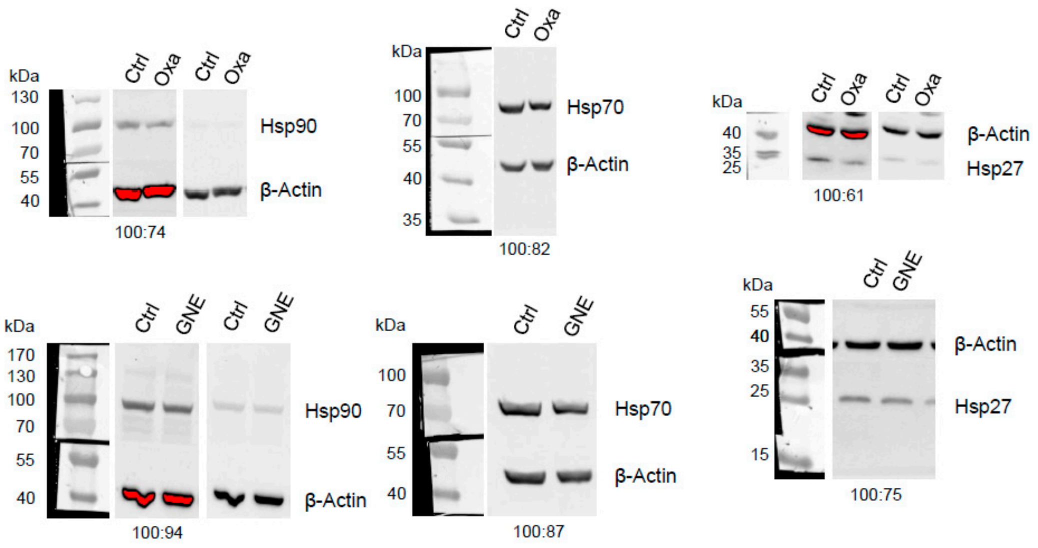

Figure S4. Western blot figures for Figure 1b. Ctrl: untreated cells, Oxa: Oxamate treated cells, GNE: GNE140 treated cells, HSP: Heat shock protein.

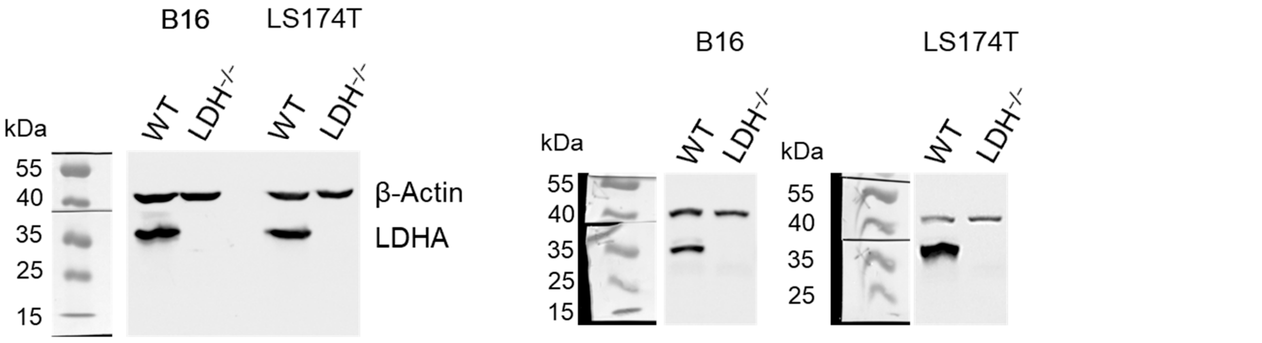

Figure S5. Western blot figures for Figure 2a. WT: wildtype, LDH$^{-/-}$: *LDHA*/*B* double knockout.

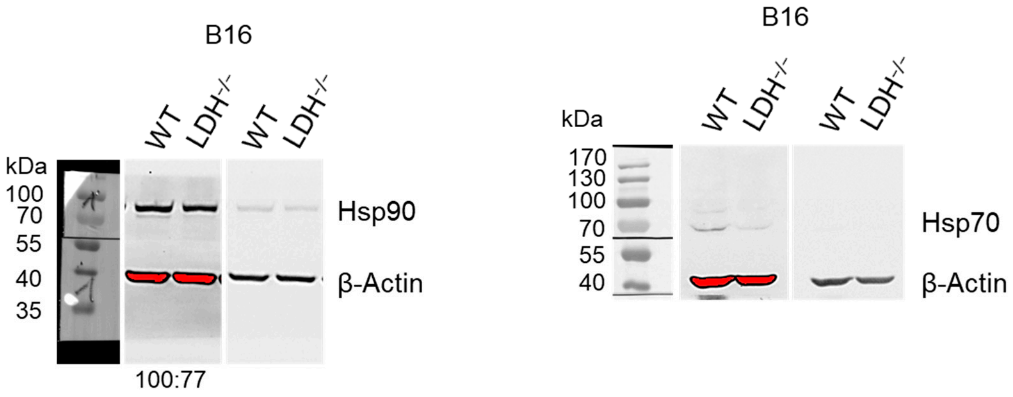

Figure S6. Western blot figures for Figure 3a. WT: wildtype, LDH$^{-/-}$: *LDHA*/*B* double knockout, HSP: Heat shock protein.

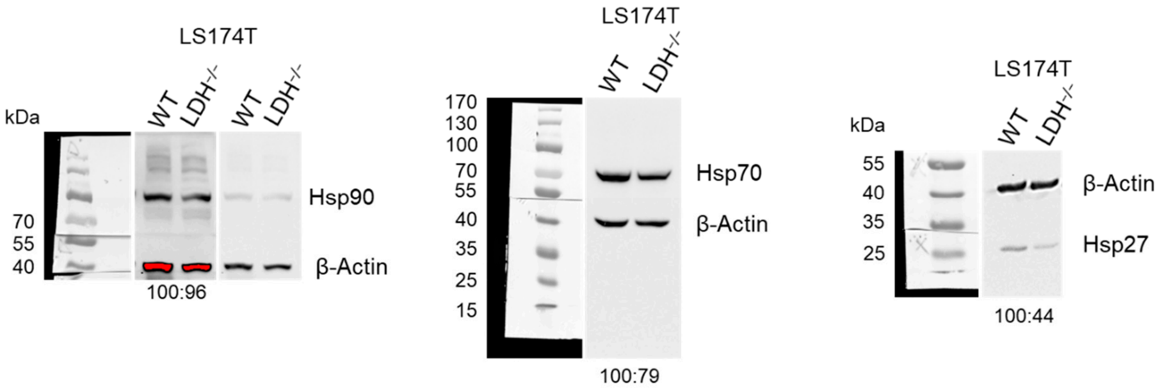

Figure S7. Western blot figures for Figure 3b. WT: wildtype, LDH$^{-/-}$: *LDHA*/*B* double knockout, HSP: Heat shock protein.

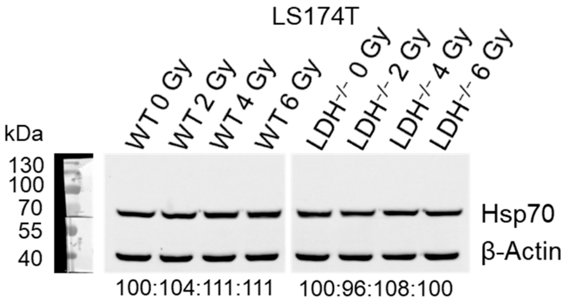

Figure S8. Western blot figures for Figure 5e. WT: wildtype, LDH: *LDHA*/*B* double knockout, HSP: Heat shock protein, Gy: Gray.

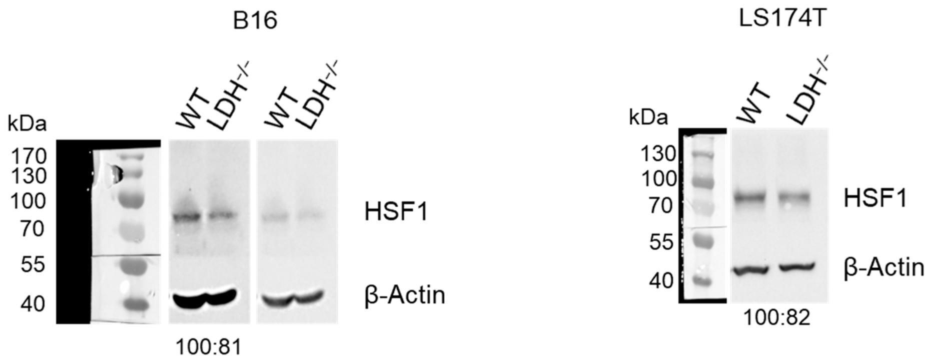

Figure S9. Western blot figures for Figure S1a. WT: wildtype, LDH$^{-/-}$: *LDHA*/*B* double knockout, HSF1: Heat shock factor 1.

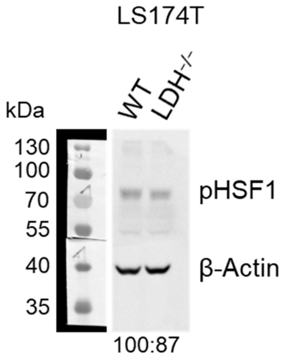

Figure S10. Western blot figures for Figure S1b. WT: wildtype, LDH$^{-/-}$: *LDHA*/*B* double knockout, pHSF1: phosphorylated heat shock factor 1.

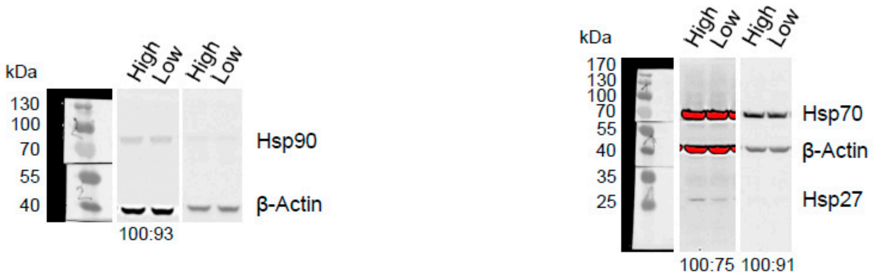

Figure S11. Western blot figures for Figure S2c. High: 4500- mg/mL glucose, Low: 1000- mg/mL glucose, HSP: heat shock protein.

Table S6. Summary of the radiobiological parameters depicted in Figure 5a–c.

**Cell Line**	**D_{50} (Gy)**[a]	**SER**[b]	**α (Gy^{-1})**[c]	**β (Gy^{-1})**[c]
LS174T				
untreated	2.27	1.00	0.23	0.03
60 mM Oxamate	1.83	1.24	0.27	0.06
WT	2.33	1.00	0.25	0.02
$LDH^{-/-}$	1.15	2.03	0.62	−0.01
B16				
WT	6.17	1.00	−0.04	0.03
$LDH^{-/-}$	3.88	1.59	0.10	0.02

[a] D_{50}: dose (Gy) required for 50% inactivation of a tumor cell population; [b] SER: Sensitizing enhancement ratio = D_{50} (control)/D_{50} (drug treatment). A SER greater than 1.20 indicates radio sensitization (indicated in bold). [c] α and β values were derived from the linear quadratic equation $f = \exp(-\alpha^*x - \beta^*x^2)$.

1. Supplementary Methods

1.1. Sample Preparation for Proteomics

Each 10 µg of protein in 0.1× RIPA buffer were subjected to tryptic digest by applying a modified filter-aided sample preparation (FASP) procedure [22, 23]. After protein reduction and alkylation using DTT and iodoacetamide, the samples were denatured in UA buffer (8 M urea in 0.1 M Tris/HCl, pH 8.5), centrifuged on a 30 kDa cut-off filter device (Sartorius, Goettingen, Germany) and washed three times with UA buffer and twice with 50 mM ammonium bicarbonate (ABC). Proteins were proteolyzed for 2 h at room temperature using 0.5 µg Lys-C (Wako Chemicals, Neuss, Germany) and, subsequently, for 16 h at 37 °C using 1 µg trypsin (Promega, Mannheim, Germany). Peptides were collected by centrifugation (10 min at 14,000 *g*) and acidified with 0.5% trifluoroacetic acid (TFA) and stored at −20 °C.

1.2. Mass Spectrometric Measurements

A LC-MSMS analysis was performed in the data-dependent acquisition (DDA) mode. MS data were acquired on a Q-Exactive HF-X mass spectrometer (Thermo Scientific, Waltham, MA, USA), each coupled online to a nano-RSLC (Ultimate 3000 RSLC; Dionex). The tryptic peptides were automatically loaded onto a C18 trap column (300 µm inner diameter (ID) × 5 mm, Acclaim PepMap100 C18 and 5 µm, 100 Å, LC Packings) at a 30 µL/min flow rate. For chromatography, a C18 reversed-phase analytical column (nanoEase MZ HSS T3 Column, 100 Å, 1.8 µm, 75 µm × 250 mm, Waters, Milford, MA, USA) at a 250 nL/min flow rate in a 95-minute nonlinear acetonitrile gradient from 3% to 40% in 0.1% formic acid was used. The high-resolution (60,000 full width at half-maximum) MS spectrum was acquired with a mass range from 300 to 1500 *m*/*z* with an automatic gain control target set to 3×10^6 and a maximum of 30 ms injection time. From the MS prescan, the 15 most abundant peptide ions were selected for fragmentation (MSMS) if at least doubly charged, with a dynamic exclusion of 30 seconds. MSMS spectra were recorded at a 15,000× resolution with the automatic gain control target set to 5×10^2 and a maximum of 50 ms of injection time. The normalized collision energy was 28, and the spectra were recorded in the profile mode.

1.3. Data Processing – Protein Identification

Proteome Discoverer (PD) 2.4 software (Version 2.4.1.15; Thermo Fisher Scientific, Waltham, MA, USA) was used for peptide and protein identification via a database search (Sequest HT search engine) against the Swiss-Prot human database (Release 2020_02, 20,349 sequences in PD) considering the full tryptic specificity, allowing for up to two missed tryptic cleavage sites, a precursor mass tolerance of 10 ppm and a fragment mass tolerance of 0.02 Da. The carbamidomethylation of Cys was set as a static modification. Dynamic modifications included the deamidation of Asn or Gln, oxidation of Met and a

combination of Met loss with acetylation on the protein N-terminus. A percolator was used for validating the peptide spectrum matches and peptides, accepting only the top-scoring hit for each spectrum and satisfying the cut-off values for FDR <1% and a posterior error probability <0.01. The final list of proteins complied with the strict parsimony principle.

1.4. Data Processing–Label-Free Quantification

The protein abundances for quantification were based on the peak intensity values of the proteotypic peptides. Normalization was performed on the total peptide amount to account for the sample loading errors. The protein abundances were calculated, summing up the single abundance values for admissible peptides. The final protein ratio was calculated using the median abundance values of four replicates for each of the experimental groups: WT and LDH$^{-/-}$. The statistical significance of the ratio change was ascertained by employing the *t*-test approach described in reference [25], which was based on the presumption that we look for expression changes for proteins that are just a few in comparison to the number of total proteins being quantified. The quantification variability of the nonchanging "background" proteins can be used to infer which proteins change their expressions in a statistically significant manner.

© 2021 by the authors. Licensee MDPI, Basel, Switzerland. This article is an open access article distributed under the terms and conditions of the Creative Commons Attribution (CC BY) license (http://creativecommons.org/licenses/by/4.0/).
